# Application of Molecular Dynamics Simulations to Determine Interactions between Canary Seed (*Phalaris canariensis* L.) Bioactive Peptides and Skin-Aging Enzymes

**DOI:** 10.3390/ijms241713420

**Published:** 2023-08-30

**Authors:** José E. Aguilar-Toalá, Abraham Vidal-Limon, Andrea M. Liceaga, Maria L. Zambrano-Zaragoza, David Quintanar-Guerrero

**Affiliations:** 1 Departamento de Ciencias de la Alimentación, División de Ciencias Biológicas y de la Salud, Universidad Autónoma Metropolitana, Unidad Lerma. Av. de las Garzas 10. Col. El Panteón, Lerma de Villada 52005, Estado de México, Mexico; j.aguilar@correo.ler.uam.mx; 2 Red de Estudios Moleculares Avanzados, Instituto de Ecología A.C. (INECOL), Carretera Antigua a Coatepec 351, Xalapa 91073, Veracruz, Mexico; 3 Protein Chemistry and Bioactive Peptides Laboratory, Purdue University, 745 Agriculture Mall, West Lafayette, IN 47907, USA; 4 Laboratorio de Procesos de Transformación y Tecnologías Emergentes de Alimentos-UIM, FES-Cuautitlán, Universidad Nacional Autónoma de México, Cuautitlán Izcalli 54714, Estado de México, Mexico; luz.zambrano@unam.mx; 5 Laboratorio de Posgrado en Tecnología Farmacéutica, FES-Cuautitlán, Universidad Nacional Autónoma de México, Av. 1o de Mayo s/n, Cuautitlán Izcalli 54714, Estado de México, Mexico; quintana@unam.mx

**Keywords:** bioactive peptides, canary seed, elastase, tyrosinase, antioxidant activity, skin-ageing, skin-aging, molecular dynamics simulations

## Abstract

Food bioactive peptides are well recognized for their health benefits such as antimicrobial, antioxidant, and antihypertensive benefits, among others. Their drug-like behavior has led to their potential use in targeting skin-related aging factors like the inhibition of enzymes related with the skin-aging process. In this study, canary seed peptides (CSP) after simulated gastrointestinal digestion (<3 kDa) were fractioned by RP-HPLC and their enzyme-inhibition activity towards elastase and tyrosinase was evaluated in vitro. CSP inhibited elastase (IC_50_ = 6.2 mg/mL) and tyrosinase (IC_50_ = 6.1 mg/mL), while the hydrophobic fraction-VI (0.2 mg/mL) showed the highest inhibition towards elastase (93%) and tyrosinase (67%). The peptide fraction with the highest inhibition was further characterized by a multilevel in silico workflow, including physicochemical descriptor calculations, antioxidant activity predictions, and molecular dynamics-ensemble docking towards elastase and tyrosinase. To gain insights into the skin permeation process during molecular dynamics simulations, based on their docking scores, five peptides (GGWH, VPPH, EGLEPNHRVE, FLPH, and RPVNKYTPPQ) were identified to have favorable intermolecular interactions, such as hydrogen bonding of polar residues (W, H, and K) to lipid polar groups and 2–3 Å van der Waals close contact of hydrophobic aliphatic residues (P, V, and L). These interactions can play a critical role for the passive insertion of peptides into stratum corneum model skin-membranes, suggesting a promising application of CSP for skin-aging treatments.

## 1. Introduction

The market for natural skin-aging prevention cosmetics and personal care products continues to increase as consumers demand more naturally derived products. In this sense, the cosmetics industry is showing interest in skin cosmetics formulated with food-derived bioactive compounds that can exert a protective and therapeutic function in the skin [1]. This group of products falls under the “nutricosmetics” category, a term that describes those nutraceuticals, i.e., substances that are a food or part of a food that provide medical or health benefits, with cosmetic applications [2].

To date, most scientific literature on food bioactive peptides with nutricosmetic applications have been reported primarily for fish, bovine, and porcine proteins [3,4,5,6]. Our research laboratory studied the anti-skin aging properties of a plant protein (chia seed) related to its inhibition of key enzymes related to skin-aging. Our in vitro studies [7] showed that the <3 kDa chia seed peptides exhibited inhibitory activities towards elastase (65.32%, IC_50_ = 0.43 mg/mL), tyrosinase (58.74%, IC_50_ = 0.66 mg/mL), hyaluronidase (26.96%, IC_50_ = 1.28 mg/mL), and collagenase (28.90%, IC_50_ = 1.41 mg/mL). These results demonstrated the potential of plant-derived proteins to generate bioactive peptides with anti-skin aging properties.

Hairless canary seed (*Phalaris canariensis* L.) is considered an emerging plant crop even though it has been used for centuries as a traditional medicine in Latin America to treat chronic diseases such as obesity, diabetes, and hypertension. In fact, hairless canary seed was granted GRAS status by the Food and Drug Administration (FDA) [8]. In addition, work from our lab on bioactive peptides from canary seeds has shown their high antioxidant activity [8], which may prove valuable for targeting the extrinsic (i.e., environmental, sun UV-ray damage) and intrinsic (i.e., natural cell or chronological aging) factors associated with skin-aging. Therefore, the aim of this study was to evaluate the in vitro effect of canary seed peptides towards skin-aging enzymes, and determine their ligand–receptor interactions at multi-level skin-aging targets. 

Bioinformatics and in silico methodologies (e.g., molecular dynamics and molecular docking simulations) are becoming widely used tools to predict potential bioactivities associated with peptides. In particular, molecular dynamics simulations (MDS) can deliver information that allows scientists to interpret functional mechanisms of proteins/peptides and other biomolecules potentially taking place in situ. For example, MDS and ensemble molecular docking analyses were used for prediction of physicochemical properties (i.e., hydrophobicity, hydrophilicity, intestinal stability, antiangiogenic, antihypertensive, and anti-inflammatory) of chia seed peptides [9]. Authors identified that five known-sequence peptides and five de novo peptides had the lowest energy score and higher affinity for ACE (angiotensin converting enzyme) and VEGF (vascular endothelial growth factor). Likewise, Gunalan, et al. [10] and Mudgil, et al. [11] reported that several peptides were docked to antihypertensive target enzymes (e.g., renin and ACE) through MDS, in order to understand their inhibition mechanisms. Similarly, using molecular docking tools, Aguilar-Toalá and Liceaga (2020) identified seven chia seed peptides involved in skin-aging enzyme–peptide pair interactions (including hydrogen bond), with most interactions occurring in the vicinity of the active site of the skin-aging enzyme elastase [7].

In this study, the in vitro and in silico behavior of canary seed peptides was evaluated towards the inhibitory properties of enzymes related to the aging process in human skin. Different bioinformatic tools were applied to understand the possible mechanism of action of the bioactive peptides, i.e., ensemble docking of key enzymes and permeability of peptides through stratum corneum membrane using conventional molecular dynamics simulations.

## 2. Results and Discussion

### 2.1. In Vitro Inhibition of Aging-Enzymes by Peptide Fractions

A total of six canary seed peptide fractions were obtained after purification using RP-HPLC (Figure S1 in Supplementary Materials). Of these eluted fractions, fraction six (F-VI) had the highest inhibitions towards tyrosinase (67%) and elastase (93%) (Table 1). This inhibitory activity is believed to be a result of the higher content of hydrophobic peptides present in latter eluted fraction (i.e., F-VI). Peptide hydrophobicity is recognized to be a relevant structural parameter related to a peptide’s biological activity [9]. This fraction (F-VI) was also reported to have potent inhibitory activity towards angiotensin converting enzyme (ACE) and pancreatic lipase [12]. Therefore, this fraction was deemed the most potent and selected for further analysis and characterization by molecular dynamics simulations.

### 2.2. Molecular Dynamics Simulations for Multi-Level Skin Aging Targets

#### 2.2.1. Identification and Prediction of Bioactive Peptides from Canary Seed

**Figure 1:**
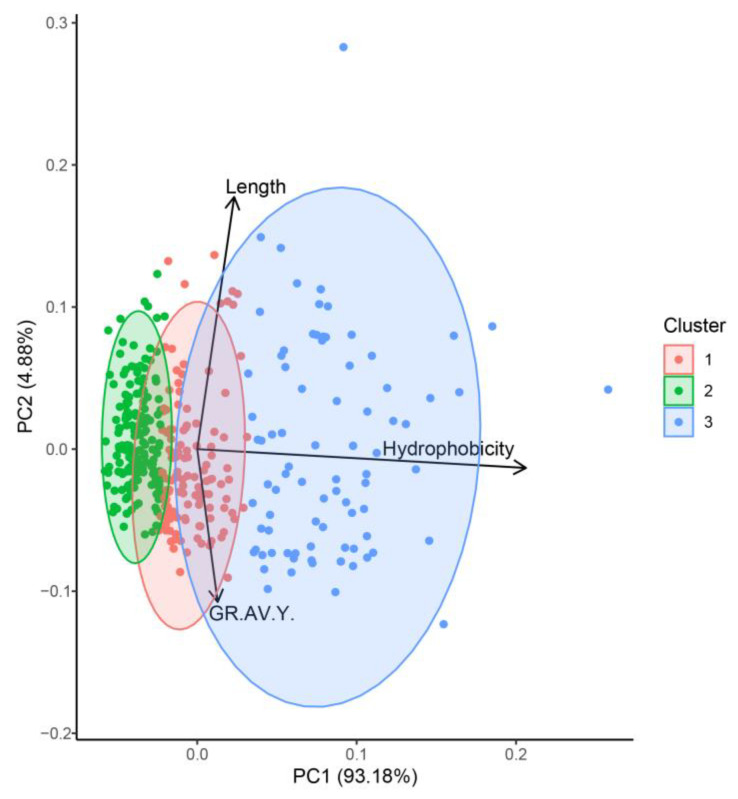
Principal component analysis and clustering of physicochemical descriptors from canary seed peptide fraction (F-VI). Eigenvalues of the covariance matrix on the principal component space. Two main components account for more than ~95% of the variation and only one linear combination of properties (cluster 3) grouped three relevant descriptors for bioactivity and their ability for penetrating cells: length, hydrophobicity, and grand average of hydropathy (GRAVY).

The potential pharmacokinetic profile of bioactive compounds can be addressed by comparison of physicochemical descriptors against well-known drugs, such as molecular weight, hydrophobicity, grand average of hydropathy, hydrogen bond acceptors, hydrogen bond donors, and peptide length. In this study, these descriptors were calculated with R Package “Peptides” v. 2.4.4) [13] and represent the first criteria for ligand-based virtual screening methodologies, which are commonly applied to a great amount of available data [14,15]. The calculated physicochemical descriptors were subjected to dimensional reduction techniques to decrease the sample complexity and hierarchize the evaluation process. From the resulting three clusters of peptides, length and hydrophobicity were grouped as more relevant physicochemical descriptors (Figure 1). Studies have reported that bioactive peptides between 2 and 20 amino acids in length have lower probability of degradation [16]. Also, the presence of hydrophobic cores can stabilize the peptide structure when interacting with hydrophobic surfaces such as cell membranes and nanomaterials [17,18,19,20,21]. Moreover, several reports have assessed that both properties are necessary for the display of secondary structures on pore-forming peptides, where segregation of hydrophobic and hydrophilic residues is crucial [22,23] for the presence of stabilizing forces derived from hydrophobic moments [24,25], and association of positively charged amino acid side chains with lipid headgroups [26,27], among others. The third cluster contained 160 canary seed peptides with the most favorable ability for penetrating cells, including length, hydrophobicity, and grand average of hydropathy (GRAVY) properties; these 160 peptides were selected for further molecular simulations (Figure 1). Several reports, including our research, have contributed to the understanding of skin-aging mechanisms that bioactive peptides can display towards different skin-aging targets [1,21,28,29]. In the present study, a multi-level simulation approach was applied consisting of prediction of antioxidant activity, molecular docking to key skin-aging enzymes, and all-atom molecular dynamics of stratum corneum membranes.

#### 2.2.2. Identification and Prediction of Canary Seed Peptides with Antioxidant Activity

The antioxidant activity is recognized as the ability to quench unpaired electron moieties, such as free radical species and highly oxidant compounds. It is highly desirable that anti-skin-aging compounds can display antioxidant activities under oxidative stress conditions. To gain insights into antioxidant activities of the bioactive peptide fraction (F-VI), the deep learning-based AnOxPePred algorithm was applied to calculate the probability of a particular sequence to present antioxidant, free radical scavenger, or quenching activities [30]. Other studies have also successfully applied AnOxPePred as a tool to predict antioxidant bioactivity of abalone [31] and egg [32] peptides. In these studies, the antioxidant scores predicted by AnOxPePred of several peptides correlated with their antioxidant activity evaluated by in vitro chemical [32] and cellular assays [31].

**Figure 2:**
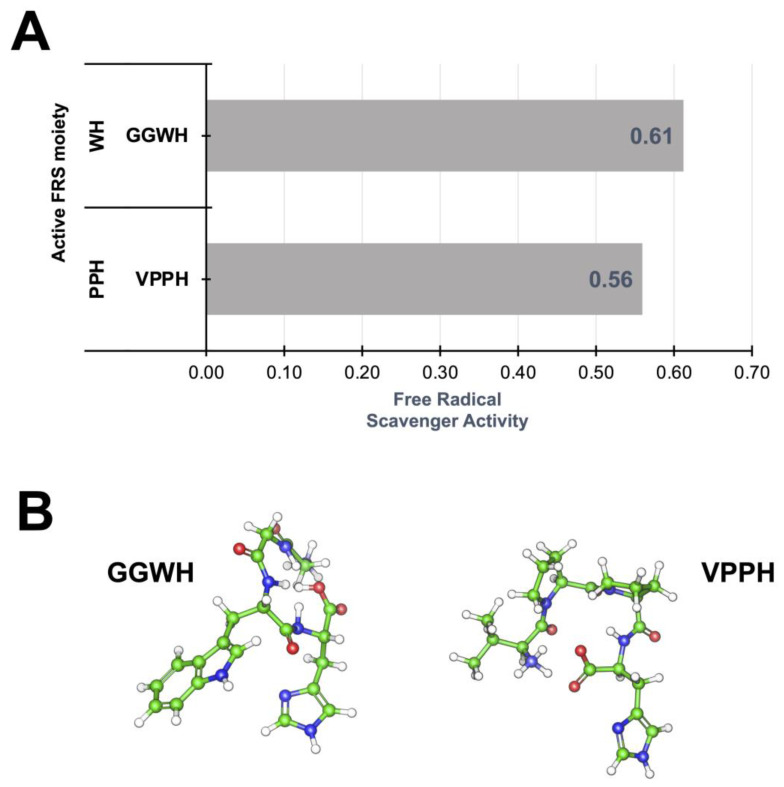
Free radical scavenging mechanism of selected canary seed peptides. (**A**) Most common free radical scavenger (FRS) structural fingerprint present in canary seed peptides. (**B**) Molecular representation of two FRS peptides (GGWH and VPPH) with the highest scavenging activity. Values calculated with AnOxiPePred [30].

The 160 peptides selected from the clustering analysis (cluster 3) in Figure 1 displayed probable free radical scavenger activity (Table S1 and Figure S2 in Supplementary Materials) with the presence of a structural amino acid fingerprint (WH and PPH), consisting of tryptophan (W), histidine (H), and proline (P) as the most common sequence moiety (Figure 2A). These sequences are related to aromatic residues that can withdraw unpaired electrons such as histidine (H) and tryptophan (W) that are common residues with high reaction rates for electron transfer [33] and, thus, radical scavenging activity. Moreover, these residues can create coordination complexes with metal ions and therefore decrease the reaction rates for electron transfer [34], albeit of residue oxidation when folding around metal centers (Figure 2B). The canary seed peptides in this study were consistent with length and hydrophobic properties designated for positive interactions with cell membranes. Furthermore, the presence of free radical scavengers associated with aromatic residues in the same peptide cluster suggest that these peptides can be effective towards inhibiting skin-aging activities.

#### 2.2.3. Bioactive Canary Seed Peptides as Inhibitors of Skin-Aging Enzymes

**Figure 3:**
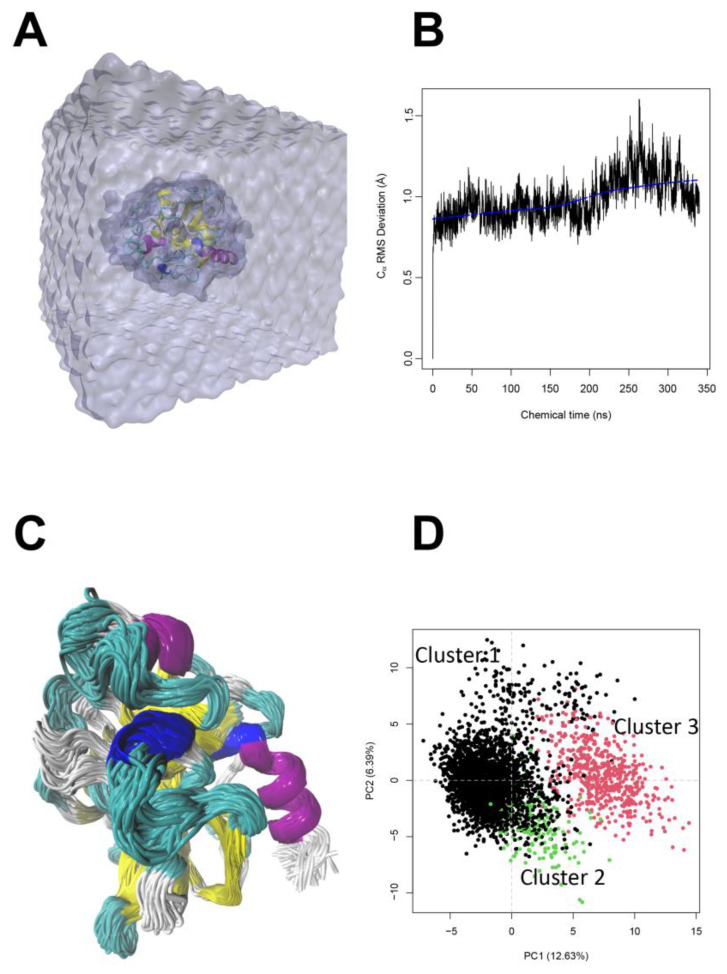
All-atom molecular dynamics simulation of the enzyme elastase. (**A**) MDS-solvated model system of elastase. (**B**) Time series evolution of alpha carbon (Cα) RMSD. The deviations were calculated for the whole enzyme but only 5 residues on C- and N-terminal groups. (**C**) Ensemble representation of elastase structures from 350 ns of MDS; the structure is colored by secondary structure elements, where purple represents an alpha-helix, cyan represents a loop, yellow shows beta strands, blue shows coils, and white represents N- or C-terminus. (**D**) Principal component analysis of elastase trajectory; three diverse clusters were calculated using Euclidean distances.

As indicated above, a total of 160 peptides with desirable physicochemical properties and free radical scavenger scores were evaluated as potential inhibitors of skin-aging related enzymes (elastase and tyrosinase) through an ensemble docking scheme. Initially, to enhance docking sampling of peptides, the crystallographic structures of the enzymes were simulated under a Gaussian Accelerated Molecular Dynamics (GaMD) sampling technique. In this method, artificial boost potentials are applied to the potential energy and dihedral angles of peptide bonds, thus promoting the exploration of protein conformational hypersurface, and displaying processes that only appear after long simulations. GaMD is widely used for enhanced sampling, improving confirmational sampling of transmembrane proteins, and drug design [35,36,37,38]. In our study, we used GaMD to help explore different conformations of the studied skin-aging enzymes that in turn helped us improve the docking score(s). For elastase, the full trajectory was sorted to retrieve statistical representative conformations with a PCA-hierarchical clustering analysis over alpha carbon (Cα) positions (Figure 3A,B). This analysis showed that selected conformations displayed differences ~0.5 Å between each other and a dispersed pattern in three well defined clusters (Figure 3C,D). For tyrosinase, the MDS system comprised a 15 Å TIP3P water molecules solvent box around the enzyme. Due to the highly flexible nature of the data (variance of Cα RMSD), hierarchical clustering analysis was used to recover the most representative structures from the trajectory (Figure S3 in Supplementary Materials). The RMSD trajectory showed that tyrosinase folding was more flexible (~1.5 Å), with a hydrophobic core around a metal center that promoted the stabilization of the residues around the binding site.

For both enzymes, the 160-peptide sequence library was evaluated against selected conformations at high Monte Carlo exhaustiveness, showing that, inside the library, peptides can be found with high affinity as denoted by consensus docking score (Table 2). We used SMINA docking package for our analysis since this is a version of a GPU-accelerated technique and it is enhanced with deep learning corrections. Furthermore, this methodology is widely used because it is capable of reproducing binding modes [39]. In the case of elastase, the presence of peptides with histidine (H) residues at a C-terminal position promote the interaction with glutamic (E79) and tyrosine (Y82), close to the Ca^2+^ binding site. In contrast, the presence of a hydrophobic core (PP, PN, PA, and LP residues) can be considered as a property that increases vdW energy and promotes stabilization inside the enzyme core. Interestingly, for tyrosinase–peptide complexes, the presence of terminal histidine and a hydrophobic core in the peptide structure promotes more stable interactions (more negative docking score). Moreover, the presence of tryptophan, histidine, and acidic amino acid residues can directly impact the affinity of the peptides towards the active site of tyrosinase, since those residues can replace a coordination group of the enzyme metal center.

#### 2.2.4. Stratum Corneum Passive Permeability of Bioactive Peptides Using Molecular Dynamics Simulations

To describe the diffusion of canary seed peptides through skin cell membranes using molecular dynamics simulations (MDS), it is essential to understand the interactions between peptide structures and the lipid bilayer components, as well as the conformational changes that occur during the permeation process. MDS are valuable tools that provide insights into the behavior of peptides in various environments, such as aqueous solutions, eutectic solvents, and lipid bilayers [40]. It is imperative to use approximate models for the lipid bilayer, due in part to the structural and dynamic nature of fluidic membranes, i.e., simple membrane models like anionic or zwitterionic micelles, or a more complex model like bacterial, plasma, or multi-layer skin membranes. The choice of the model can significantly affect the organization and dynamics of the lipid membranes. In our study, the membrane model systems were based on the stratum corneum membranes reported by Badhe, et al. [41], which was subjected to MDS (Table 3). For example, Badhe et al. showed that the impact of specific ceramide concentrations on the lipid matrix, evaluated with neutron diffraction methods and MDS, can shed light on molecular phenomena that influence lipid layer packing. Other studies used steered MDS to predict the membrane permeation process of cyclic peptides across lipid bilayers [42]. However, understanding lipid composition and fluidic behavior is fundamental to recognize diffusional processes.

**Figure 4:**
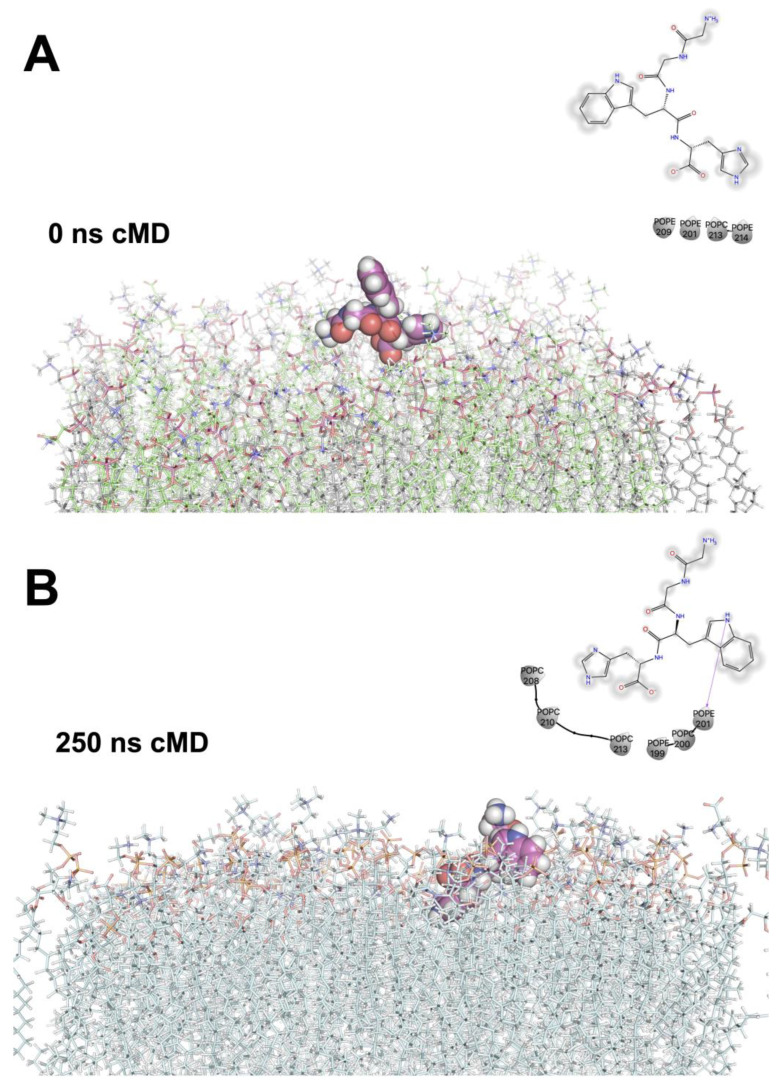
All-atom molecular dynamics simulation of the tetra-peptide GGWH complexed to a stratum corneum skin model depicting the peptide’s structural trajectory. (**A**) Initial structure at 0 ns. (**B**) Final structure at 250 ns of peptide–skin membrane complex.

In the present study, short (0 to 250 ns) molecular mechanics simulations of selected peptides complexed to stratum corneum membranes showed that hydrogen bond interactions between positively charged residues (H, W, or K) is a part of the onset of diffusional processes (Figure 4A,B). Our data reveal that, before the partitioning of the peptides to the inter-membrane space, stable hydrogen bonds (~3.5 Å) should form between lipid polar-head moieties, such as phosphoethanoamine (POPE), phosphocholine (POPC), or phosphoserine (POPS), and peptide charged residues. Subsequent steps include hydrophobic interactions between aliphatic residues like proline, alanine, valine, and glycine with long fatty acid chains in the lipid environment and conformational changes that promote the close vdW interactions (~2 Å from aliphatic carbon atoms). The onset of the previous steps has implications for drug distribution, membrane protein folding, and the energetics of voltage gating channels [43], since partitioning from water to non-polar environments is an energetically demanding process.

The molecular dynamics simulations of the tetra-peptide GGWH are a representative example of the proposed steps for the passive diffusion processes described before. The fully solvated peptide sequence (GGWH) was initially positioned 3 Å away from the stratum corneum model. From the initial 20 ns, the azole moiety oriented to POPC or POPE and established hydrogen bonds between the H-azole sidechain and the phosphatidic moiety. These interactions induced conformational changes on the peptide, measured as alpha-carbon RMSD ~4 Å. The conformational changes displayed (~250 ns of molecular dynamics simulations) showed that the surrounding lipids were able to form a cavity in the exposed surface accessible to solvents (Figure 4B). The tryptophan (W) residue, which is both aromatic and hydrophobic, appeared to move towards the aliphatic portion of the lipids, where tightly packed ceramides and cholesterol were located within the membrane. Tight lipid packaging is critical for sterol displacement by ceramides, as demonstrated by the comparison of the sterol-displacing abilities.

When peptides include lysine (K) residues in their sequence, the hydrogen bond and subsequent partitioning effect inside the membranes are faster (~100 ns). However, due to the highly flexible sidechain of K, the stabilization of this moiety inside lipid membrane takes longer and is highly depended on the lipid environment (i.e., the presence of cholesterol residues that improve packing of aliphatic groups). The formation of secondary hydrogen bonds could promote the partition of the peptide core inside the membranes. Moreover, it can also increase the solvation energy of the peptide since hydrogen bond interactions are established between atoms with polarity differences, which in turn can form hydrogen bonds with the solvent.

## 3. Materials and Methods

Hairless canary seeds (CDC Cibo) were purchased from a commercial vendor (Can-pulse Foods LTD, Saskatoon, SK, Canada). Alcalase^®^ (protease from *Bacillus licheniformis*) was purchased from Novozymes (Bagsvaerd, Denmark). Elastase enzyme (from porcine pancreas, Type IV), and N-(methoxysuccinyl)-Ala-Ala-Pro-Val p-nitroanilide, Tyrosinase enzyme (from mushroom) were purchased from Sigma-Aldrich (St. Louis, MO, USA). All chemicals used were reagent grade and generally obtained from VWR International (Radnor, PA, USA) or Thermo Fisher Scientific (Waltham, MA, USA).

### 3.1. Preparation of Canary Seed Peptides

Peptides were prepared according to method of Urbizo-Reyes, Liceaga, Reddivari, Kim and Anderson [12]. Briefly, defatted canary seed powder was diluted (22.5 mg of protein/mL) and homogenized in deionized water using a Sorvall Omni Mixer with a macro-attachment assembly (Norwalk, CT, USA). The pH was adjusted to 8.0 with 2 M NaOH and proteolysis was carried out for 4 h at 50 ± 2 °C using 3% (*w*/*w*) Alcalase^®^. Proteolysis was terminated by heating at 95 ± 2 °C for 15 min. After the solution was cooled down, it was centrifuged at 17,636× *g* for 15 min (Avanti J-26S Centrifuge, Beckman-Coulter Inc., Brea, CA, USA) and the supernatant was collected as soluble canary seed peptides (CSP) and stored at −80 °C until use.

CSP were then subject to simulated gastrointestinal digestion (SGD) [12]. Briefly, a peptide solution (10 mg of protein/mL) was first adjusted to pH 2.0 using 1 M HCl and incubated with pepsin (4% *w*/*w* of protein) at 37 °C for 2 h. Then, 0.9 M NaHCO_3_ was used to adjust pH to 5.3, and 1.0 M NaOH to further increase the pH to 7.5. Finally, pancreatin was added (4% *w*/*w* of protein) and the mixture was incubated at 37 °C for 2 h. Digestion was terminated by pasteurization (95 ± 2 °C) for 15 min. The solution was cooled to room temperature and centrifuged (11,000× *g* for 15 min); the supernatant was collected and referred to as digested peptides. Preliminary studies showed that small peptides (<3 kDa) from canary seed were the most biologically active [8]. Therefore, peptides were fractionated by ultrafiltration using a <3 kDa cutoff membrane and referred to as canary seed peptides after simulated gastrointestinal digestion (CSP-SGD). CSP-SGD were frozen at −80 °C for 12 h and freeze-dried using a Labconco FreeZone Plus 2.5 L cascade benchtop freeze dry system (Labconco Corp., Kansas City, MO, USA). The peptide content was normalized using the bicinchoninic acid (BCA) protein assay (Thermo Scientific, Rockford, IL, USA) before subsequent purification.

CSP-SGD were further purified using Reverse-Phase High Performance Liquid Chromatography (RP-HPLC), as previously described [12], using a Waters 2690 HPLC system (Waters Corporation, Milford, MA, USA), equipped with an automatic sample injector and 2998 UV photodiode array (PDA) detector. Briefly, CSP-SGD were suspended in HPLC-grade deionized water and filtered (0.45 µm). The HPLC was injected with 50 µL CSP-SGD (7 mg protein/mL) at 25 °C into an XBridge™ BEH130 C18 column (10 μm, 10 × 150 mm, Waters Inc., Milford, MA, USA) using a two-stage gradient procedure: starting with an eluent concentration ratio of 100:0% (*v*/*v*), mobile phase A (deionized water with 0.1% (*v*/*v*) TFA) to mobile phase B (acetonitrile with 0.1% (*v*/*v*) TFA), decreasing to 75:25% (*v*/*v*) A:B over 35 min, and then to 60:40% (*v*/*v*) A:B over 60 min, at a flow rate of 0.3 mL/min. Elution of peptide fractions was monitored at a wavelength of 280 nm. Eluted peptide fractions (F1–F6) were collected, concentrated by lyophilization, and stored at −80 °C until analysis.

### 3.2. Evaluation of In Vitro Anti-Aging Bioactive Properties

#### 3.2.1. Elastase Inhibition Assay

The inhibition of the enzyme elastase was evaluated as described by Aguilar-Toalá and Liceaga [7]. Briefly, 100 µL of <3 kDa (2.5, 5.0, and 10 mg/mL) and RP-HPLC (0.2 mg/mL) peptide fractions or buffer (control) were combined with 50 µL of substrate 10 mM/of N-(methoxysuccinyl)-Ala-Ala-Pro-Val p-nitroanilide (10 mM), and incubated for 15 min at 37 °C. Solutions were mixed with 50 μL of pre-incubated (5 min, 37 °C) elastase (50 mU) and allowed to react for 15 min. The percentage of inhibition was calculated by recording the absorbance at 405 nm, using Equation (1), where *OD_control_* and *OD_sample_* are the optical density of the control and samples, respectively:

(1) $$\left(\%\right)\;Inhibiiton=\frac{OD_{control}-OD_{sample}}{OD_{control}}\times 100$$

#### 3.2.2. Tyrosinase Inhibition Assay

Tyrosinase inhibition was evaluated according to Aguilar-Toalá and Liceaga [7]. Aliquots (100 µL) of peptide fraction (0.2 mg/mL) or buffer-control were combined with 50 µL substrate (10 mM 3,4-dihydroxy-L-phenylalanine) and incubated for 15 min at 30 °C. The solutions were then mixed with 50 μL of pre-incubated (5 min, 30 °C) tyrosinase (150 U), and the reaction was carried out for 15 min. Absorbance of the reaction was recorded at 450 nm and the percent inhibition determined using equation [1]. The half-maximal inhibitory (IC_50_) was used to determine the potency of the samples towards each skin-aging enzyme (elastase and tyrosinase). Calculations were determined at three different peptide concentrations (2.5, 5.0, and 10 mg/mL) of the <3 kDa fraction.

#### 3.2.3. Identification of Elastase and Tyrosinase Inhibitory Peptides

The most active peptide fraction (F-VI) was further selected for peptide sequence identification by LC–MS/MS in the Proteomics Core Facility at the Indiana University School of Medicine (Indianapolis, IN, USA) as previously described by Urbizo-Reyes, Liceaga, Reddivari, Kim and Anderson [12]. The peptide fraction (200 µg) was suspended in 100 µL of formic acid (0.1% *v*/*v*) and 10 µL of the resulting solution was injected into a 5 cm trap column and an EasySpray (801A) column (15 cm, 2 µm particle size, 50 µm diameter) on an UltiMate 3000 HPLC and Q-Exactive Plus (ThermoFisher Scientific, Waltham, MA, USA) mass spectrometer. Data were analyzed using PEAKS Xpro software (Bioinformatics Solutions Inc., Waterloo, ON, USA).

### 3.3. Molecular Dynamics Simulations for Multi-Level Skin Aging Targets

#### 3.3.1. Prediction of Physicochemical Descriptors from Bioactive Peptide Fractions

The potential grand average of hydropathy (GRAVY), isoelectric point (pI), length, and molecular weight were predicted using peptides sequences with R package “peptides” v 2.4.4 [13]. All CSP 3D structures were modeled with OPLS-AA forcefield inside Schrödinger Maestro Suite v.2020-4 (New York, NY, USA). Theoretical isolectric point for each peptide was calculated using pKa values from the EMBOSS database. The GRAVY values were calculated by adding the hydropathy value for each residue and dividing by the length of the sequence. Principal component analysis (PCA) and hierarchical clustering analysis (HCA) were applied to physicochemical descriptors to identify possible clusters that correlated with bioactivity.

#### 3.3.2. Prediction of Physicochemical Descriptors from Bioactive Peptide Fractions

Prediction of antioxidant activity such as free radical scavengers and chelation properties for all peptide sequences were calculated with the convolutional neural network algorithm implemented in AnOxPePred v 1.0 (http://services.bioinformatics.dtu.dk/, accessed on April 2023) [30].

#### 3.3.3. Model Preparation of Skin-Aging Related Enzymes

The starting protein coordinates of the two skin-aging enzymes (elastase and tyrosinase) were retrieved from the X-ray structure (PDB code: 1LVY [44] and 5M6B [45], respectively). Both structures were prepared and minimized by adding the missing loops and side chains with Modeller V 10.1. The OPLS-AA forcefield from Schrödinger Maestro 2020-4 suite (New York, NY, USA) was applied to remove the explicit solvent molecules and for the addition of hydrogen atoms to each conformer.

#### 3.3.4. Ensemble Docking of Skin-Aging Enzymes

Molecular dynamics simulations (MDS) were performed with pmemd.cuda [46] of the Amber20 package (https://ambermd.org/, accessed on April 2023) [47,48]. Initial minimization (combined steepest descendent and conjugated gradient scheme) and simulated annealing were carried out under the NvT ensemble. The simulated annealing protocol was carried out in six consecutive steps. The entire system was heated for 300 ps at 10 K, 100 K for 1000 ps, 300 K for 1000 ps, 400 K for 300 ps, 400 K for 500 ps, and finally cooled to 300 K for 2000 ps. A linear interpolation between two adjacent time points was employed. NpT ensemble was used for pre-equilibration (50,000 ps) and 300 ns (conventional molecular dynamics) at constant pressure (1 atm) with Monte Carlo Barostat = 2. The Particle Mesh Ewald (PME) method was employed to compute long-range electrostatic interactions (1 × 10^−9^ of tolerance) in the periodic systems at integration time of 2 fs. Weak coupling was used to apply the temperature to an external bath temperature coupling at 298 °K. The alpha carbon RMSD values were calculated with CPPTRAJ v5.0 [49].

GaMD is an enhanced sampling technique designed to smooth biomolecular potential energy surfaces and reduce energetic barriers between the different processes in biomolecular systems [50]. This method adds a harmonic boost potential to dihedral angles as well as directly to potential energy. A more detailed description of the methodology has been described in previous studies [51,52,53]. GaMD productions of 350 ns were achieved with sigma0P = 6.0, sigma0D = 6.0.

Binding modes and score calculations (Vinardo scoring function [54]) were performed with Smina v1.2 [55] for Ensemble docking of 160 peptides. All the peptides were previously optimized at PM6 level of theory with MOPAC 2016 (http://OpenMOPAC.net, accessed on April 2023). The overall search parameters were 0.375 Å mesh step, 64 exhaustiveness value, and a maximum of 25,000 poses per evaluation. The orthorhombic search space dimensions were set to 25 × 25 × 25 Å centered on each enzyme-binding site. All scores were approximated using the consensus scoring approximation per binding site [56]. Finally, based on docking scores, the top-five (GGWH, VPPH, EGLEPNHRVE, FLPH y RPVNKYTPPQ) ranked peptides were selected for further analysis below.

#### 3.3.5. Molecular Dynamics Simulations of Stratum Corneum–Peptide Complexes

A lineal heating scheme was applied to a model lipid bilayer model of stratum corneum. A total of 297 lipids, 15,000 explicit water molecules, and the top-five ranked peptides (GGWH, VPPH, EGLEPNHRVE, FLPH y RPVNKYTPPQ) were simulated at pH 7 and 310 °K. All peptides were aligned to the normal *z*-axis of membranes. Minimization, heating, and equilibration steps were applied as previously described for elastase and tyrosinase. However, the membrane systems (Table 3) in complex with ensemble docking top ranked peptides were equilibrated at 310 °K for 50 ns and extended during 250 ns for production stages at 2 fs the integration timestep. The webserver CHARMM-GUI web server [57] and LIPIDS 21 forcefield [58] were applied to describe lipid topology and parameters. The lipid order values, area per lipid, lipid anchor, and peptides’ alpha carbon RMSD values were calculated with CPPTRAJ v5.0 [49].

## 4. Conclusions

Canary seed peptides have potential to be used as inhibitors towards enzymes related to skin-aging. In vitro analysis showed that canary seed peptide fraction F-VI contained bioactive peptides capable of inhibiting tyrosinase (67%) and elastase (93%). Virtual screening of the peptides showed that grand average of hydropathy, hydrophobicity, and length are relevant physicochemical properties to consider for these potential bioactive peptide fractions. The presence of anti-free radical fingerprints in the peptides suggests that peptide fraction (F-VI) can promote anti-skin aging activity by a combination of properties, ranging from the scavenging of free radical species to direct inhibition of enzymes involved in skin-aging, as determined by molecular docking simulations. Furthermore, to probe that those bioactive peptides can reach their molecular targets in the skin tissue, molecular dynamics simulations were applied to the five top-ranked peptide sequences (GGWH, VPPH, EGLEPNHRVE, FLPH and RPVNKYTPPQ), which showed that initial hydrogen bonds and partitioning of the hydrophobic cores of peptides are the governing intermolecular interactions for passive insertion of the peptides into skin membranes. Molecular dynamics simulations of the tetra-peptide (GGWH) complexed to a stratum corneum skin model also showed that surrounding lipids in the skin membrane were capable of forming a surface cavity, while aromatic and hydrophobic residues were able to migrate towards the lipids’ aliphatic portion. To enhance the prediction of skin-aging peptides, further simulations can be performed with a combination of different peptide fractions to assess the aggregation phenomena resulting from hydrophobic interactions.

Due to the increasing market for natural skin-aging prevention cosmetics and personal care products, this study demonstrates that canary seed peptides can potentially be used as natural, anti-aging ingredients. Bioinformatic tools such as molecular dynamics simulations can be useful in predicting the behavior of these bioactive compounds towards skin-aging enzymes. Our findings suggest a promising application of canary seed peptides for the development of skin-aging treatments in cosmetic/cosmeceutical products, which could slow down the intrinsic skin-aging processes and contrast the extrinsic ones.

## Figures and Tables

**Table 1:** Skin-aging enzyme inhibition by canary seed peptides derived from the <3 kDa SGD and RP-HPLC fractions, respectively.

Peptide Fraction	Elastase Inhibition (%) ^1^	Tyrosinase Inhibition (%) ^1^
<3 kDa (2.5 mg/mL)	33.76 ± 0.012	29.95 ± 0.011
<3 kDa (5 mg/mL)	45.96 ± 0.018	54.19 ± 0.040
<3 kDa (10 mg/mL)	65.26 ± 0.008	62.63 ± 0.034
IC_50_	6.24 mg/mL	6.10 mg/mL
F-I	ND	ND
F-II	ND	ND
F-III	ND	ND
F-IV	84.05 ± 0.002	14.96 ± 0.004
F-V	55.14 ± 0.004	ND
F-VI	92.82 ± 0.001	66.93 ± 0.001

^1^ The % inhibition was determined at 2.5, 5, and 10 mg/mL of the <3 kDa simulated gastrointestinal (SGD) fraction and at 0.2 mg/mL of the RP-HPLC fractions (F-I to F-VI). The % Inhibition are average values of triplicate determinations ± standard deviation. ND = inhibition not detected. The half-maximal inhibitory (IC_50_) was used to determine the potency of the samples towards each skin-aging enzyme. Calculations were determined at the three different <3 kDa peptide concentrations (2.5, 5.0, and 10 mg/mL).

**Table 2:** Ensemble docking of canary seed peptides towards two skin-aging enzymes (tyrosinase and elastase).

Tyrosinase		Elastase	
Peptide	Docking Score ^1^	Peptide	Docking Score ^1^
GGWH	−9.36 ± 0.56	VPPH	−8.00 ± 0.85
VPPH	−9.18 ± 1.22	EGLEPNHRVE	−7.92 ± 0.91
FLPH	−9.08 ± 0.75	GGWH	−7.70 ± 1.01
VPHGAP	−8.94 ± 1.10	FGPAGHT	−7.32 ± 0.99
WAGW	−8.62 ± 1.24	RPVNKYTPPQ	−7.24 ± 0.72
MPYN	−8.54 ± 1.38	FLPH	−7.12 ± 1.38
ELHPQ	−8.54 ± 1.48	LLPH	−7.00 ± 1.83
FVPH	−8.46 ± 1.02	LHPE	−6.92 ± 1.24
EGLEPNHRVE	−8.44 ± 1.23	VVPH	−6.90 ± 1.16
LLPH	−8.40 ± 1.26	VPPAH	−6.86 ± 0.81
VYPN	−8.30 ± 0.92	MPYN	−6.84 ± 1.55
FHPQ	−8.26 ± 1.05	NEEWPR	−6.80 ± 0.62
LTPH	−8.20 ± 0.83	VPHGAP	−6.78 ± 1.42
FGPAGHT	−8.20 ± 0.85	FVPH	−6.74 ± 1.31
NEEWPR	−8.14 ± 0.99	WGPALH	−6.68 ± 1.30
VVPPGVPY	−8.04 ± 1.09	WAGW	−6.66 ± 1.14
WGPALH	−7.98 ± 0.99	LTPH	−6.64 ± 0.91
VPPAH	−7.86 ± 0.87	KGGCEHEV	−6.64 ± 0.53
VPPHQQ	−7.78 ± 0.66	ELHPQ	−6.56 ± 1.09
KGGCEHEV	−7.30 ± 0.66	FHPQ	−6.50 ± 1.28
PLGH	−6.94 ± 1.01	VVPPGVPY	−6.50 ± 0.85
NYPVG	−6.52 ± 0.44	VYPN	−6.44 ± 1.07
GHDPK	−6.38 ± 0.13	HTHL	−6.36 ± 1.91
CPPLH	−6.34 ± 0.62	PLGH	−6.06 ± 0.51
LHPE	−6.26 ± 0.47	VPPHQQ	−5.86 ± 0.63
SLHPQ	−6.14 ± 0.90	QAHPK	−5.76 ± 0.62
RPVNKYTPPQ	−6.10 ± 0.33	CPPLH	−5.74 ± 0.52
QAHPK	−5.98 ± 0.44	NYPVG	−5.68 ± 0.42
HTHL	−5.68 ± 0.44	SLHPQ	−5.44 ± 0.59
VVPH	−5.66 ± 0.50	GHDPK	−5.24 ± 0.38

^1^ All units are expressed as kcal · mol^−1^. Docking score values ± standard deviation were averaged from five replica conformations and evaluated with Vinardo Scoring function at exhaustiveness = 64.

**Table 3:** Lipid composition of the stratum corneum skin model bilayers per upper and lower leaflet.

Lipid ^1^	Upper Leaflet	Lower Leaflet
CER (Ceramides [NP] shingonoids)	29	30
CHOL (cholesterol)	35	35
POPC (1,2-dioleoyl-sn-glycero-3-phosphocholine)	25	27
POPE (1,2-dioleoyl-sn-glycero-3-phosphoethanolamine)	28	29
POPS (1,2-dioleoyl-sn-glycero-3-phospho-L-serine)	30	29

^1^ Lipid composition of model stratum corneum as reported by Badhe et al. [30].

## Data Availability

The data presented in this study are available on request from the corresponding author.

## Supplementary Materials

The following supporting information can be downloaded at: https://www.mdpi.com/article/10.3390/ijms241713420/s1.
