# Supplementary material for: Application of Molecular Dynamics Simulations to Determine Interactions between Canary Seed (Phalaris canariensis L.) Bioactive Peptides and Skin-Aging Enzymes
Source: Int J Mol Sci. 2023 Aug 30;24(17):13420. doi: 10.3390/ijms241713420 (PMC10487734; doi:10.3390/ijms241713420)
Supplement: Supplementary file 1 [file ijms-24-13420-s001.zip › ijms-2543010-supplementary.pdf]

Supplementary Materials

Application of molecular dynamics simulations to determine interactions between canary seed (*Phalaris canariensis* L) bioactive peptides and skin-aging enzymes

J.E. Aguilar-Toalá [1]; Abraham Vidal-Limon [2]*; Andrea M. Liceaga [3]*; M. Zambrano Zaragoza[4]; and D. Quintanar-Guerrero[5]

[1] Departamento de Ciencias de la Alimentación, División de Ciencias Biológicas y de la Salud, Universidad Autónoma Metropolitana, Unidad Lerma. Av. de las Garzas 10. Col. El Panteón, Lerma de Villada 52005, Estado de México, Mexico; j.aguilar@correo.ler.uam.mx

[2] Red de Estudios Moleculares Avanzados, Instituto de Ecología A.C. (INECOL), Carretera Antigua a Coatepec 351, Xalapa 91073, Veracruz, Mexico; abraham.vidal@inecol.mx

[3] Protein Chemistry and Bioactive Peptides Laboratory, Purdue University, 745 Agriculture Mall, West Lafayette, IN, 47907, USA; aliceaga@purdue.edu

[4] Laboratorio de Procesos de Transformación y Tecnologías Emergentes de Alimentos-UIM, FES-Cuautitlán, Universidad Nacional Autónoma de México, Cuautitlán Izcalli, Estado de México 54714, Mexico; luz.zambrano@unam.mx

[5] Laboratorio de Posgrado en Tecnología Farmacéutica, FES-Cuautitlán, Universidad Nacional Autónoma de México, Av. 1o de Mayo s/n, Cuautitlán Izcalli, Estado de México 54714, Mexico; quintana@unam.mx

* Correspondence: (AVL) abraham.vidal@inecol.mx; (AML) aliceaga@purdue.edu; Tel.: +1 -765-496-2460

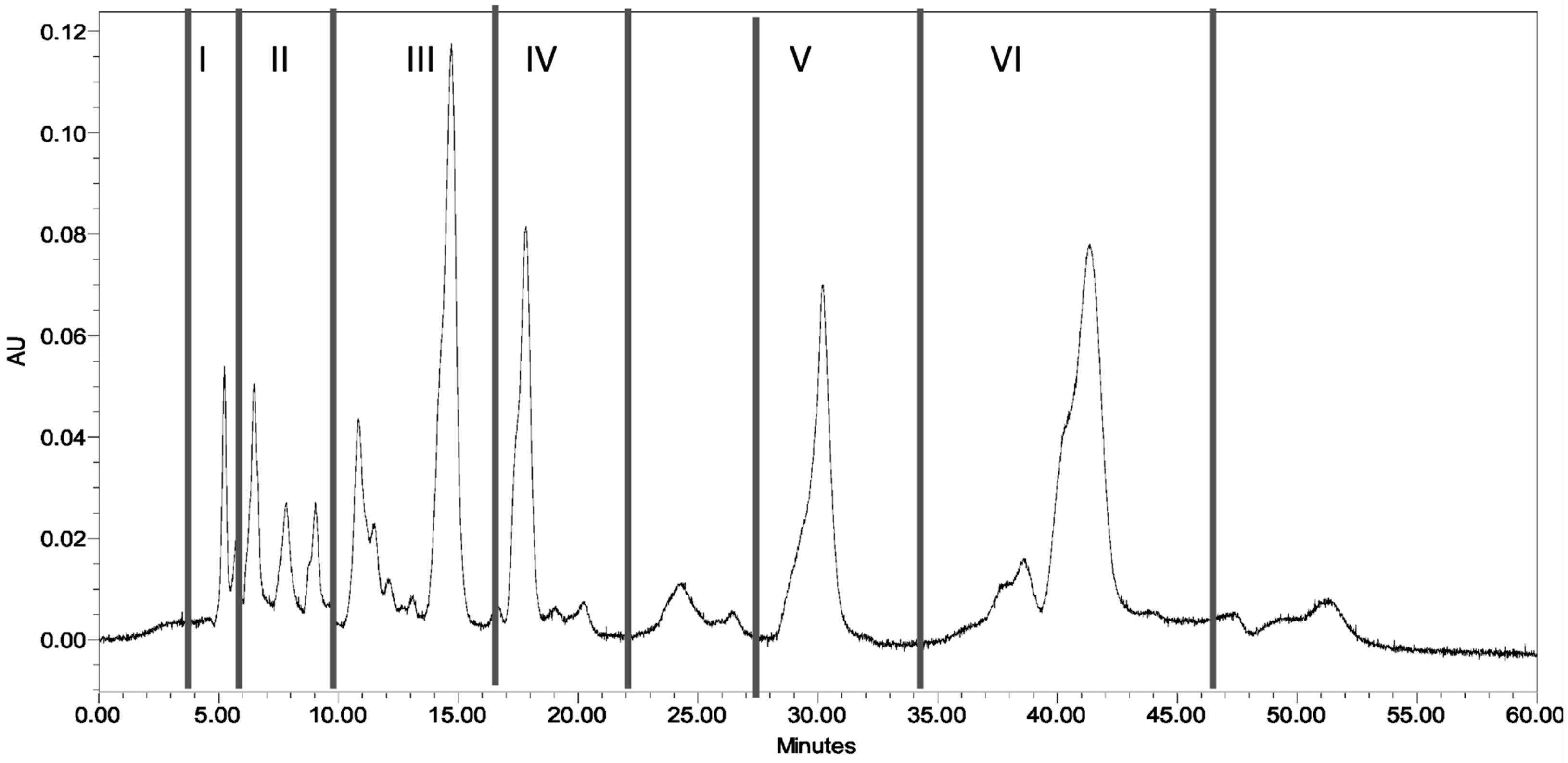

Figure S1. RP-HPLC fractionations (F1-F6) derived from the <3 kDa gastrointestinal digestion of canary seed peptides.

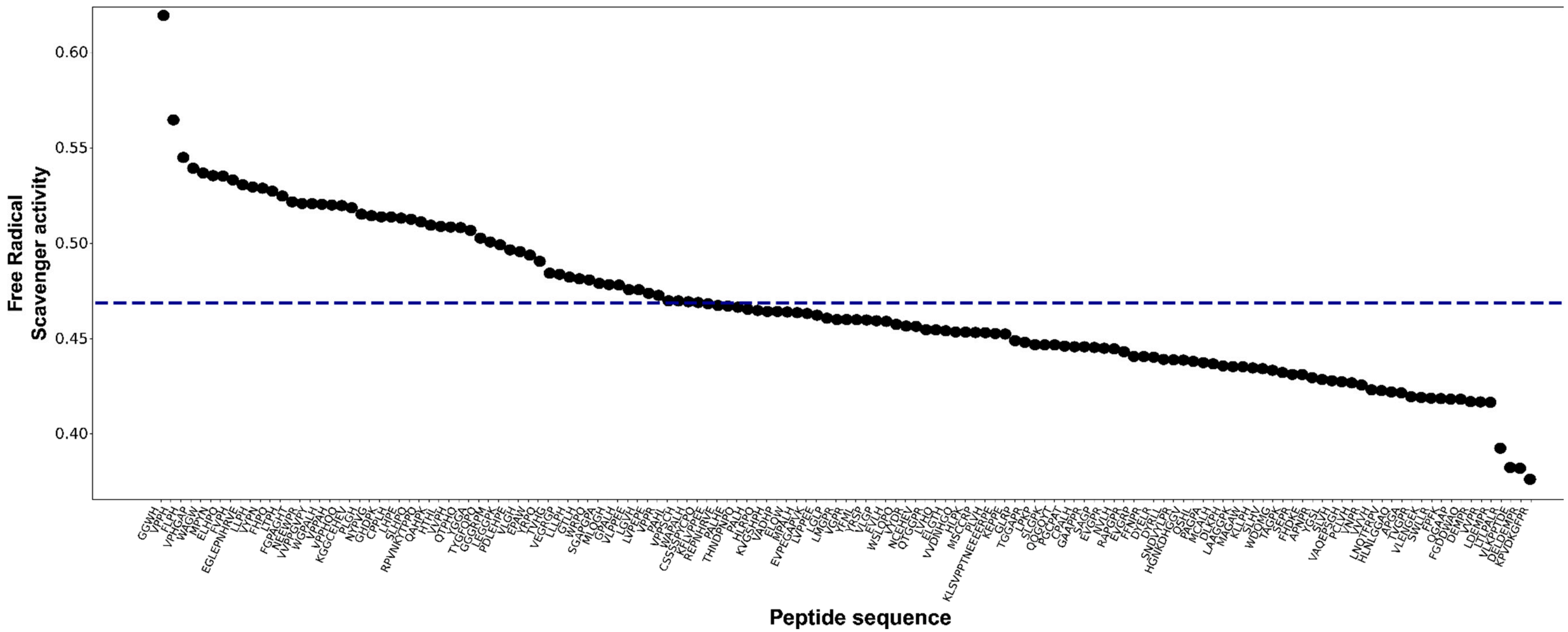

Figure S2. Antioxidant susceptibility, where Free Radical Scavenger (FRS) was calculated with AnOxiPePred [30].

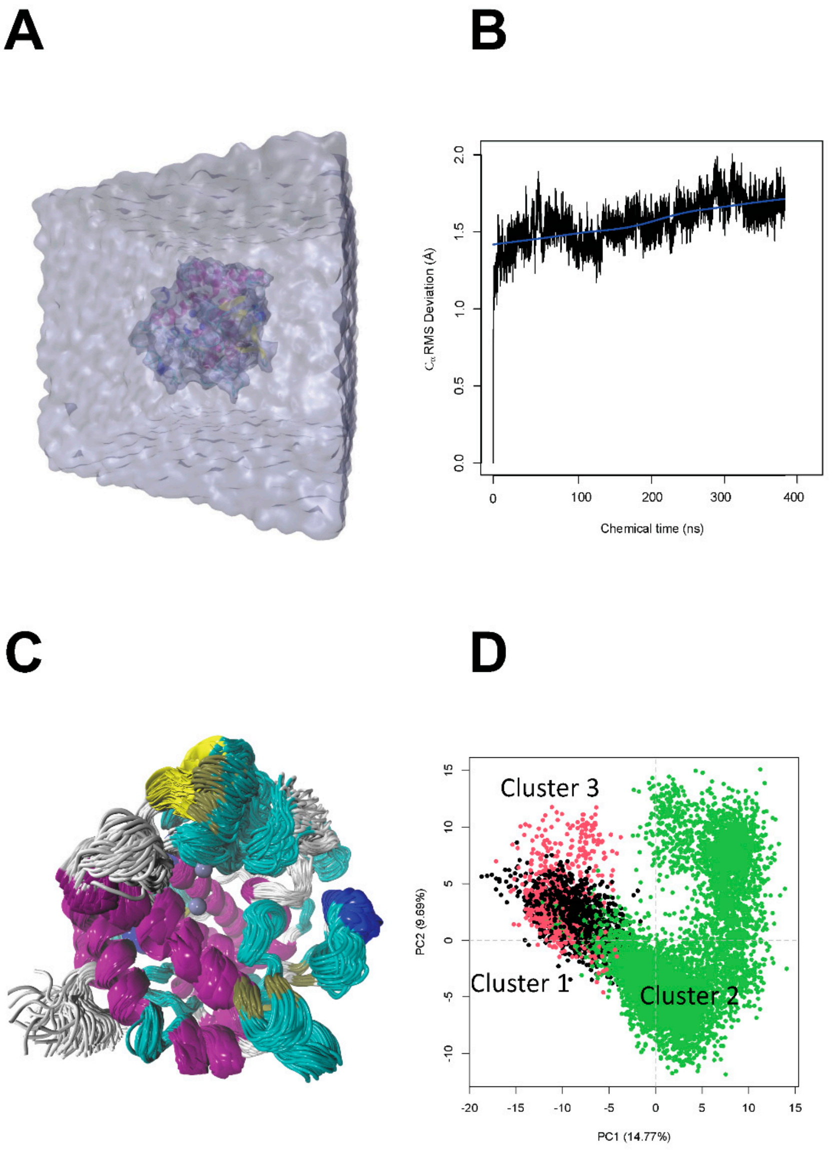

Figure S3. All-atom Molecular Dynamics Simulation of the enzyme tyrosinase. (A) MDS solvated model system of tyrosinase. (B) Time series evolution of alpha-C RMSD. The deviations were calculated for the whole enzyme but 5 residues on C- and N-terminal groups. (C) Ensemble representation of tyrosinase structures from 350 ns of MDS, the structure is colored by secondary structure elements: purple, alpha helix; cyan, loop; yellow, beta strand; blue, coils; white, N or C terminus. (D) Principal component analysis of elastase trajectory; three diverse clusters were calculated using Euclidean distances.

Table S1. Free radical scavenging mechanism of selected canary seed peptides calculated with AnOxiPePred [30].

Free Radical Scavenger activity	Peptide Sequence	Active FRS moiety
0.6120	GGWH	WH
0.6120	GGWH	WH
0.5592	VPPH	PPH
0.5402	FLPH	PH
0.5348	VPHGAP	PH
0.5323	WAGW	WAGW
0.5310	MPYN	YN
0.5310	MPYN	YN
0.5308	ELHPQ	ELH
0.5288	FVPH	PH
0.5288	FVPH	FVPH
0.5264	EGLEPNHRVE	EGLE-NHR
0.5252	LLPH	PH
0.5246	VYPN	VY
0.5231	FHPQ	FHP
0.5207	LTPH	LTPH
0.5178	FGPAGHT	FGPAGHT
0.5169	NEEWPR	NEEWPR
0.5168	VVPPGVPY	VVPPGVPY
0.5165	WGPALH	WGPALH
0.5162	VPPAH	VPPAH
0.5158	VPPHQQ	VPPHQQ
0.5148	KGGCEHEV	KGGCEHEV
0.5115	PLGH	PLGH
0.5107	NYPVG	NYPVG
0.5102	GHDPK	GHDPK

0.5101	CPPLH	CPPLH
0.5095	LHPE	LHPE
0.5089	SLHPQ	SLHPQ
0.5076	RPVNKYTPPQ	RPVNKYTPPQ
0.5059	QAHPK	QAHPK
0.5053	HTHL	HTHL
0.5050	VVPH	VVPH
0.5047	QTPHQ	QTPHQ
0.5033	YGGA	YGGA
0.4994	TYGFQPQ	TYGFQPQ
0.4975	GGGRPM	GGGRPM
0.4960	LEGGPK	LEGGPK
0.4935	PDLEHPE	PDLEHPE
0.4925	VLGH	VLGH
0.4908	EPAW	EPAW
0.4908	EPAW	EPAW
0.4877	YRPQ	YRPQ
0.4817	TVHG	TVHG
0.4811	VEGRGP	VEGRGP
0.4797	LLLPH	LLLPH
0.4788	GTLH	TLH
0.4788	GTLH	GTLH
0.4782	WRPQ	WRPQ
0.4765	SGAPGPA	SGAPGPA
